# New Insights on Expandability of Pre-Cured Epoxy Using a Solid-State CO_2_-Foaming Technique

**DOI:** 10.3390/polym13152441

**Published:** 2021-07-24

**Authors:** Uy Lan Ngoc Du, Christian Bethke, Volker Altstädt, Holger Ruckdäschel

**Affiliations:** 1 Department of Polymer Engineering, University of Bayreuth, Universitaetsstrasse 30, 95447 Bayreuth, Germany; Du.Uy-Lan@uni-bayreuth.de (U.L.N.D.); christian.bethke@uni-bayreuth.de (C.B.); altstaedt@uni-bayreuth.de (V.A.); 2 Bavarian Polymer Institute and Bayreuth Institute of Macromolecular Research, University of Bayreuth, Universitaetsstrasse 30, 95447 Bayreuth, Germany

**Keywords:** solid-state foaming, epoxy foam, CO_2_ batch foaming, storage modulus

## Abstract

Foaming an epoxy is challenging because the process involves the curing reaction of epoxy and hardener (from monomer to oligomer, to a gel and a final three-dimensional crosslinked network) and the loading of gas phase into the epoxy phase to develop the cellular structure. The latter process needs to be carried out at the optimum curing stage of epoxy to avoid cell coalescence and to allow expansion. The environmental concern regarding the usage of chemical blowing agent also limits the development of epoxy foams. To surmount these challenges, this study proposes a solid-state CO_2_ foaming of epoxy. Firstly, the resin mixture of diglycidylether of bisphenol-A (DGEBA) epoxy and polyamide hardener is pre-cured to achieve various solid-state sheets (preEs) of specific storage moduli. Secondly, these preEs undergo CO_2_ absorption using an autoclave. Thirdly, CO_2_ absorbed preEs are allowed to free-foam/expand in a conventional oven at various temperatures; lastly, the epoxy foams are post-cured. PreE has a distinctive behavior once being heated; the storage modulus is reduced and then increases due to further curing. Epoxy foams in a broad range of densities could be fabricated. PreE with a storage modulus of 4 × 10^4^–1.5 × 10^5^ Pa at 30 °C could be foamed to densities of 0.32–0.45 g/cm^3^. The cell morphologies were revealed to be star polygon shaped, spherical and irregularly shaped. The research proved that the solid-state CO_2_-foaming technique can be used to fabricate epoxy foams with controlled density.

## 1. Introduction

Thermoset foams have become more and more essential in various industry sectors. These foams are used in applications requiring good thermal stability, high flame resistance, burning without drips and excellent solvent resistance, for example, in automotive, electronic, marine, aeronautical and space products [1,2,3]. Common thermoset foams are polyurethane, phenolic and epoxy. Even though polyurethane has widespread uses in civil engineering, transportation and furnishings, health effects also have to be considered as isocyanate exposure during processing causes irritation of the skin and mucous membranes, chest tightness and breathing difficulties [4,5,6]. Phenolic foams are less common because of their brittleness and the harmful effect of the residual formaldehyde used for curing [7]. Epoxy foams, on the other hand, do not possess these drawbacks. Moreover, epoxy foams could provide various moduli from viscoelastic to rigid foams by changing the epoxy–hardener ratio from a stoichiometric to adduct system [8]. In current practice, epoxy foams are produced in a one-stage process combining foaming and curing from viscous resins to final solid foams. The process is called the resin direct foaming process, and is a common practice for polyurethane, phenolic and epoxy. The process involves both foaming (cell nucleation, cell growth and cell stabilization) and curing (early cured, gel and fully cured). In the technical view, the fabrication of epoxy foam involves the loading of gas phases into the resin mixture, and a cell stabilization process via the curing reaction of epoxy and hardener. The foaming process depends significantly on the type of blowing agent and the rheological behavior of the epoxy–hardener mixture. Curing is essential to stabilize the cell structure. The curing reaction of epoxy and hardener increases the viscosity of the epoxy matrix and helps to reduce the diffusion of gas and to develop the cellular structure, and also to prevent gas loss by cell rupture until curing is completed. Hence, it is difficult to control the foaming window throughout the curing process and possible failures during foaming, such as coalescence and cell collapse [9]. In the resin direct foaming process, coalescence occurs when the cells grow and break their viscous border, leading to a large merging cell. Due to the environmental concerns, many types of physical blowing agent (PBA), such as low boiling hydrocarbon solvents and chlorofluorocarbons, and chemical blowing agents (CBA), such as azodicarbonamide, are prohibited [10]. 

To avoid these drawbacks, expanding microspheres and microhollow spheres are used to control the cell size and to produce syntactic foams. The syntactic foams depend greatly on the dispersion of hollow spheres, which is known as sphere-packing. This results in syntactic foams having a limited density range. Glass microsphere (GmSs) and phenolic microspheres (PhmSs) are common materials but are costly. Moreover, GmSs lead to an increase in viscosity, leading to inferior dispersion quality and hence resulting in inhomogeneous foams [11,12,13,14]. The production of glass hollow spheres involves sulfur gas, which is a toxic gas, while PhmSs also contain toxic formaldehyde [15]. Thus, the fabrication of epoxy foam needs to be carried out with new approaches.

Epoxy and hardener in the resin stage have low molecular weight, so the usage of gas blowing agents (GBAs) such as CO_2_ is inconvenient for foaming because of its high diffusion rate. In general, foaming using a GBA is only applied for thermoplastics, which have a suitably high molecular weight. Common GBAs are CO_2_ and N_2_ with different molar mobilities. CO_2_ and N_2_ are usually absorbed into solid thermoplastic polymers using a high-pressure autoclave, called a batch-foaming process, or impregnated in molten thermoplastics in an extrusion-foaming process [16]. In a batch-foaming process, foaming takes place at a suitable temperature, such as close to the melting temperature of semi-crystalline thermoplastic or above the glass transition temperature of amorphous thermoplastic; this is to activate or increase the viscoelastic behavior of the polymer chains so that they can be foamed. The polymer structure must also have sufficiently high molecular weight or long chain branches to provide superior foamability. A chain extender is normally added to increase the molecular weight of semi-crystalline thermoplastics and make them foamable [17]. The viscosity of thermoplastics can be varied by temperature and shear to optimize the foaming process [18]. 

In contrast, the viscosity of epoxy resin increases via curing and could reach similar ranges to those of thermoplastics. This leads to the research question of whether a pre-cured epoxy (preE) with an adequate viscosity or modulus range can be foamed with a GBA. It is noted that this modulus range is significantly lower than that of the fully cured epoxy, the modulus of which is normally around 2–3 GPa. In general, there are two types of preE. First, preE before the gel point containing large oligomers and, second, preE after the gel point with one network with some free reactive end groups and large *M_c_*. PreE will not melt but gel and solidify once the curing process is completed. Therefore, the term modulus is used and is more suitable to check the foamability of preE. According to thermoset rheology, a gel point is determined from the cross section of the storage modulus and loss modulus. Before the gel point, the loss modulus is higher than the storage modulus and the preE’s behavior is largely viscous and has a high damping factor (tan delta > 1). After the gel point, the preE will exhibit elastic- and plastic-dominated behavior.

Recently, CO_2_ has been applied to fabricate epoxy foam. For indirect usage, CO_2_ is captured by the amine group of polyamine hardener to produce a dual function carbamate, which is used to foam and cure epoxy resin [19]. In another case, the CO_2_ is used directly via absorption and desorption (similar to CO_2_ batch foaming) [20,21]. To implement this technique, a solid epoxy needs to be prepared in advance. A solid epoxy could be obtained from different pre-curing stages and achieve different crosslink densities. To obtain the solid-state behavior of pre-cured epoxy, the molecular weight between crosslinks (*M_c_*) is the characteristic value. According to Ito et al. (2014), CO_2_ bubble nucleation in a batch physical foaming process could be possible or impossible depending on the *M_c_* values. Their study used three different bisphenol-A diglycidyl ether epoxy (DGEBA) samples with molecular weights of 370 g/mol, 470 g/mol and 900 g/mol; the hardener used was 2-ethyl-4-methylimidazole [20]. The study reported that only the DGEBA of 900 g/mol could be foamed, while the others could not be foamed or cracked. Nevertheless, according to the viscoelastic results, a foaming temperature of 90 °C was quite close to the T_g_ of the DGEBA of 900 g/mol (T_g_ ~ 108 °C) and very low compared to the T_g_ of epoxy with 470 g/mol (T_g_ ~ 135 °C) and 370 g/mol (T_g_ ~ 170 °C). This raises the question of whether the foaming process could be carried out if the foaming temperature is near to the T_g_ of epoxy with 470 g/mol and 370 g/mol. Another study reported that a pre-curing degree from 37.7% to 46.3% was the proper foaming range for the foaming process of DGEBA and m-xylylenediamine (MXDA) [21]. The processes of absorption and releasing of CO_2_, as well as the foaming and the post-curing, were carried out in a closed vessel. Therefore, it was not clear whether the preE entered the solid stage and whether the foaming happened during the depressurizing process or not.

This research introduces an innovative solid-state CO_2_-foaming method to produce epoxy foams. The technique implies that the epoxy will be foamed at its solid state, which is completely different from common foaming epoxy, which is in a curing resin state. The main aim of this research is to understand the fundamental relationship between the molecular crosslink network and CO_2_ absorption–desorption process. Bisphenol-A diglycidylether (DGEBA) and a long chain polyamide hardener (Ancamide 260A) were used to provide a crosslinked network [22], which could assist the foaming ability. Ancamide 260A, derived from diethylene-triamine (DETA) and a fatty acid, has six H-actives from two primary amines and two secondary amines. Hence, it can react with DGEBA at room temperature or a high temperature and produce various preEs with different storage moduli and loss moduli, which behave with different foamabilities. The density of epoxy foams was defined by the *M_c_* of the preE and its modulus at a foaming temperature. 

## 2. Materials and Methods

### 2.1. Materials

**Scheme 1:**
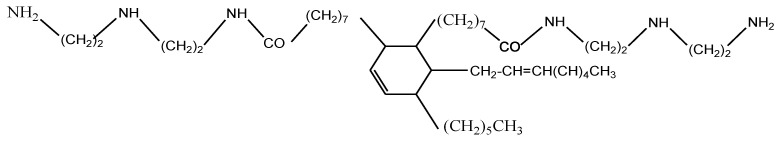
Chemical structure of Ancamide polyamide 260A.

Epoxy resin (DGEBA-DER331) was purchased from Olin (Clayton, MI, USA). DER 331 has an epoxide equivalent weight of 182 g/eq, and viscosity at 25 °C of 11–14 Pas. Ancamide 260A (a polyamide hardener) was provided by Evonik (Essen, North Rhine-Westphalia, Germany). Ancamide 260A has an equivalent weight per H active of 110, and viscosity at 25 °C of 35–45 Pas. The CO_2_ cylinder was supplied by Riesner Gase (Lichtenfels, Germany). The chemical structure of the Ancamide polyamide is shown in Scheme 1 (Sea et al., 2015).

### 2.2. Methods 

#### 2.2.1. Fabrication of Epoxy Foam by Using Solid-State CO_2_ Foaming Technique

Preparation of preE: The stoichiometric weight ratio of DGEBA and 260A was 100:60.44. A 14 g mixture of DGEBA and 260A was prepared by using a DAC 150 speed mixer (Hauschild, Hamm, Germany) at 3000 rpm for 1 min. The epoxy mixtures were casted into a mold and hot-pressed (using the PW 20 P/O/Weber, Remshalden, Germany) to obtain a sheet with dimensions of 100 mm × 100 mm × 1 mm (this was also the thickness from the rheology measurement). Silicone paper was used on both surfaces for an easier demolding process. The hot-pressing was conducted at 30 bars. The compression temperatures and times were based on the rheology results, as shown in Table 1, so that the preE could achieve the storage moduli of 20 kPa, 30 kPa and 40 kPa. The sheets were cut into 25 mm × 25 mm × 1 mm dimensions for the CO_2_ absorption step. Three specimens of 20 mm × 10 mm × 1 mm were used for checking *M_c_* via a swelling test. In addition, the completely cured epoxy was hot-pressed at 100 °C for 150 min for the calculation of the interaction parameter between epoxy and acetone. The weight uptakes of acetone of the preE and completely cured epoxy were tested. 

CO_2_ absorption: PreE with dimensions of 25 mm × 25 mm × 1 mm was used for the CO_2_ absorption. The specimens were separated using a soft mesh fabric to prevent stacking and inserted into a Berghof HR-700 autoclave. The CO_2_ absorption was carried out at a pressure of 55 bar and temperature of 25 °C for 5 h. After the given sorption time, the autoclave was depressurized from 55 bar to atmospheric pressure within 2 min. The absorbed samples were kept in a freezer at –10 °C for the free expansion process.

Free expansion in oven: Foaming temperature was varied from 40 °C to 100 °C. An oven (Memmert) with a see-through window was used. The CO_2_–epoxy plates were placed in the oven in an aluminum pan. The expansion over time was observed to identify the suitable foaming time and temperature. 

**Scheme 2:**
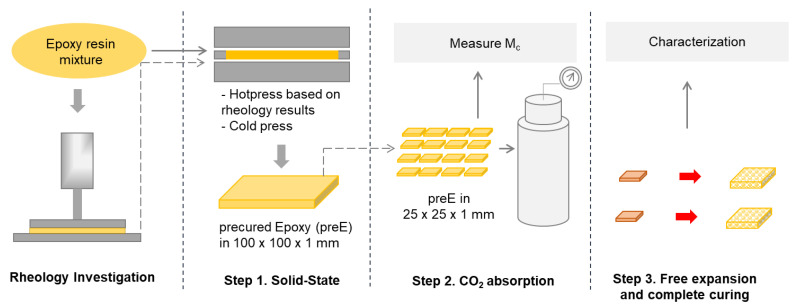
Workflow of solid-state CO_2_ foaming technique.

Post-curing process: Further curing to achieve full curing were performed in a conventional oven (Memmert ULE 400). The temperature was set to 50 °C (based on the DSC results for further curing temperature). The Workflow of solid-state CO_2_ foaming technique is shown in Scheme 2.

#### 2.2.2. Characterization and Testing

a. Rheological behavior of DGEBA and polyamide

The rheological behavior of epoxies was studied using an Anton Paar MCR301 rheometer (Graz, Austria) equipped with a 25 mm parallel plate. The sample gap between the parallel gap and the aluminum tray was set at 1 mm. First, the isothermal tests at the studied temperatures (60 °C, 70 °C and 80 °C according to DSC result) were conducted until the force limitation. All heating conditions were carried out with ω = 1 rad/s and γ = 3%. From the rheology curve, the times for the preE to achieve storage moduli of 20 kPa, 30 kPa and 40 kPa were identified. Second, the combination of isothermal and dynamic testing was implemented. The sample was tested isothermally and at fixed time to achieve the studied modulus, and then allowed to cool down at a rate of 10 °C/min to 30 °C (to obtain the storage modulus of preE at 30 °C as St.M_30 °C_). After that, the samples were heated up to 100 °C at a rate of 10 °C/min (to check the modulus of preE with temperature).

b. PreE properties

T_g_ and degree of cure: The curing behavior of preE was studied using differential scanning calorimetry (Mettler Toledo DSC1 Star System, Columbus, OH, USA). The scanning was conducted from −20 °C to 200 °C under nitrogen (N_2_) environment with a flowrate of 50 mL/min and heating rate of 10 °C/min. The glass transition temperature (T_g_) and onset temperature (T_onset_) of further curing and degree of cure were recorded. 

Crosslink density of the preE using a swelling method: The gel fraction was measured using samples of 20 mm × 10 mm × 1 mm. The gel fraction was calculated based on the weight of partly cured epoxy after soaking in acetone at 23 °C for 7 days and subsequent drying at 135 °C until the weight remained constant (*W_g_*). The gel fraction was calculated as the ratio *W_g_* and the initial weight *W_0_* after curing (Equation (1)). The crosslink density was calculated according to Equations (2) and (3) [20].

(1) $$gel\;fraction\approx \frac{W_{g}}{W_{0}}$$

(2) $$M_{C}\approx \frac{\rho V_{ace}\left(2v_{2}^{\frac{1}{2}}-v_{2}\right)}{v_{2}^{2}\left(1-2\chi \right)}$$

(3) $$v_{2}=\frac{\rho_{ace}}{\rho_{ace}+\rho \left(w_{a}-1\right)}$$

*v_2_*: volume fraction of cured epoxy in acetone-swollen sample; *ρ* and *ρ_ace_*: density of completely cured epoxy and acetone of 1.116 g/cm^3^ and 0.78 g/cm^3^, respectively; *V_ace_*: the molar volume of acetone of 74.0 cm^3^/mol; *w_a_*: saturation % of acetone uptake of cured epoxy; *χ*: interaction parameter between epoxy and acetone.

*χ* was determined from the *M_c_* and the weight uptake of acetone of completely cured epoxy. The *M_c_* of completely cured epoxy was determined from the rubbery plateau modulus (*G^0^_N_*) at a temperature of 200 °C by using an Anton Paar MCR301 rheometer (Equation (4)). The test was conducted with a 25 mm parallel plate at a frequency of 1 Hz and a heating rate of 10 °C/min with a strain of 0.05%. The mixture of DGEBA–260A was added and cured at 100 °C for 150 min, then the temperature was swept to 200 °C and held at 200 °C for 30 min.

(4) $$M_{C}\approx \frac{3\rho RT}{G_{N}^{0}}$$

The *G^0^_N_* at a temperature of 200 °C was 8475 kPa so the *M_c_* of the completely cured epoxy was 1553.94 g/mol. The completely cured epoxy had a weight uptake of acetone of 115.2%. Therefore, the *χ* was calculated to be 0.458.

CO_2_ dissolution of the preE: The weights of preEs before and after the CO_2_ absorption were measured. They are denoted as *W*_0_ and *W*_CO_*2*__, respectively. The percentage of dissolved CO_2_ was calculated by using Equation (5).

(5) $${\mathrm{CO}}_{2}\;\%=\frac{W_{{\mathrm{CO}}_{2}\;}-W_{0}}{W_{0}}\times 100\%$$

c. Properties of epoxy foam:

Density of the epoxy foams was measured by Archimedes’ principle by using the AG245 analytical balance from Mettler Toledo (Columbus, OH, USA) equipped with the density kit. 

Morphology of epoxy foams was observed by a SEM—Jeol JSM-6510 (Tokyo, Japan). To avoid charging during SEM observation, samples were sputtered with a 13 nm gold layer by using a Cressington Sputter Coater 108auto (Cressington Coating systems, Dortmund, Germany). 

## 3. Results and Discussion

### 3.1. Rheology Analysis of Epoxy—Ancamide 260A and PreE

**Figure 1:**
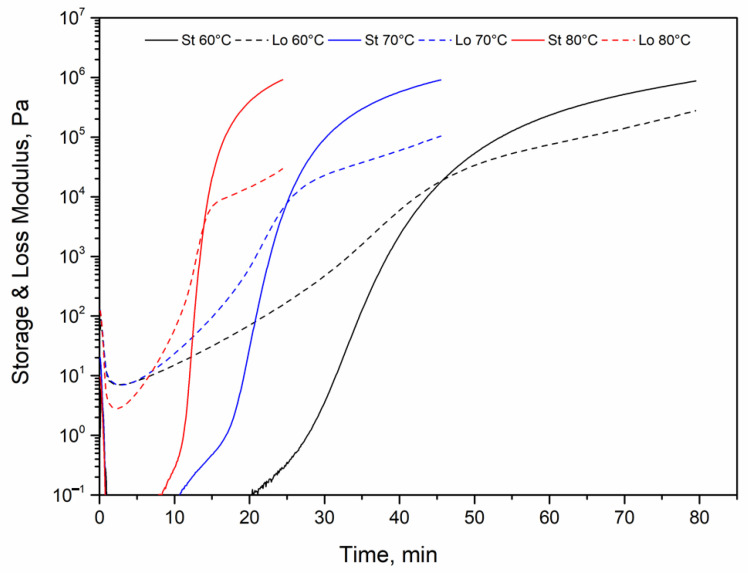
Rheology of DGEBA–260A cured at 60 °C, 70 °C and 80 °C.

The rheology curve exhibited the phase transition of epoxy resin to the gel stage and to the solid phase. It was important in this study to identify the gel point. The rheological test was used to measure the storage modulus and loss modulus of curing epoxy. The storage modulus is a measure of elasticity of the curing epoxy and its ability to store energy. The loss modulus is the ability to dissipate energy and energy lost as heat. An epoxy network develops and grows during curing, and hence increases its elasticity and storage modulus. The storage modulus increases and overtakes the loss modulus. The crossing point of both moduli is a gel point. A measurement of a gel point is carried out with the tan delta value, which is the ratio of the loss modulus over the storage modulus [23]. Since Ancamide 260A is a higher molecular weight hardener, the reaction of DGEBA and Ancamide 260A formed large molecular weight oligomers. Hence, both storage and loss moduli increased gradually at curing temperatures of 60 °C and 70 °C. In contrast, a steep increase was observed at an elevated temperature of 80 °C, as shown in Figure 1. This behavior was totally different from the rheology of DGEBA and a lower molecular weight hardener such as N-aminoethylpiperazine, (AEP) or isophorone diamine (IPDA) (Bethke et al., 2019), in which the modulus just increased slightly before the gel point and sharply increased at the gel point. The gradual increment in the modulus of 260A and DGEBA is induced by the structure of 260A, as displayed in Scheme 1. Ancamide 260A has a total of eight H-actives from two primary amines (4 H), two secondary amines (2 H) and two amide groups (2 H). The reaction of the primary amine and epoxy group of DGEBA results in chain extension (oligomers) and a new secondary amine. Further reactions between the secondary amine and epoxy ring form branched and crosslinked networks. Due to steric hindrance, further crosslinking reaction take place more slowly and need more energy to activate. Therefore, preEs fabricated from DGEBA and 260A could be controlled to achieve various specific moduli and also different pre-crosslinked network structures. A higher temperature induces faster curing, because the epoxy and polyamide are more mobile and activated. PreEs with lower *M_c_* are produced. The solid state of preE or a solid-like character is also important for the application of 3D-printable epoxy inks [24].

**Figure 2:**
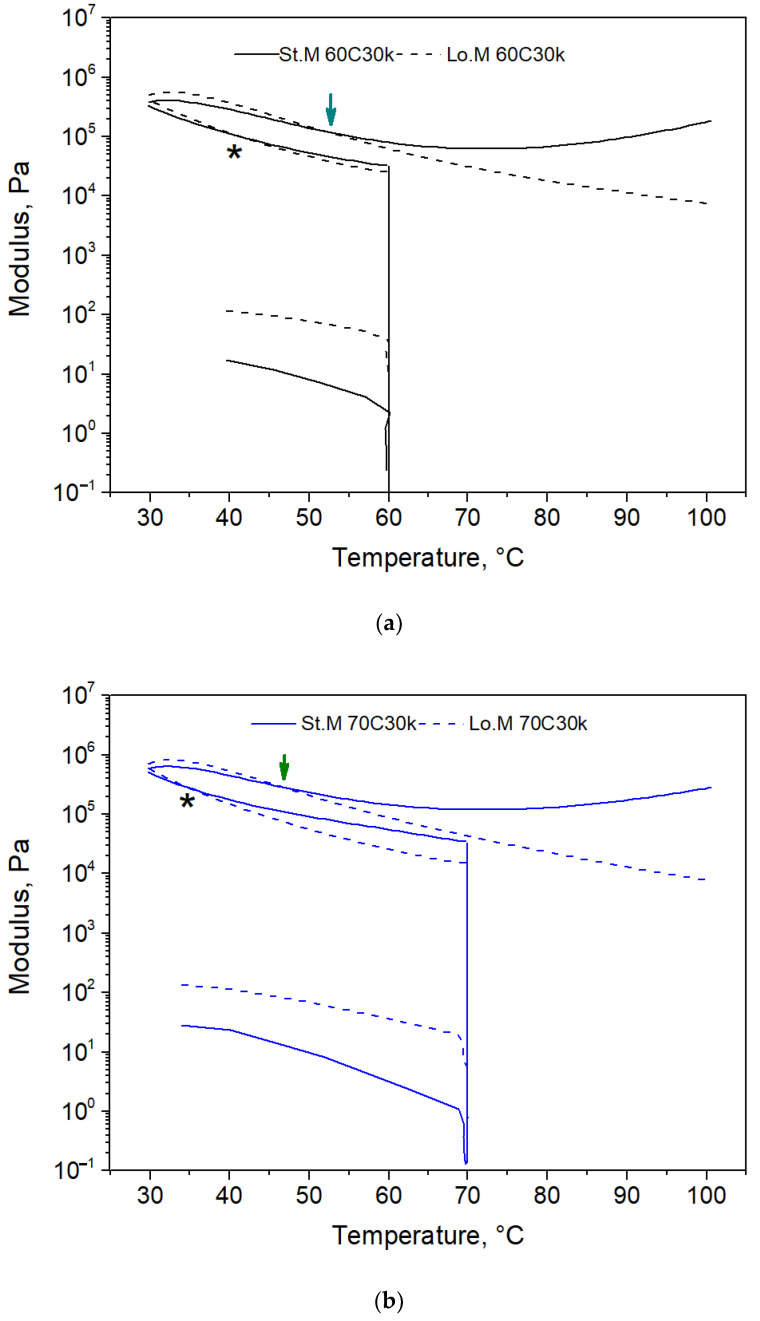

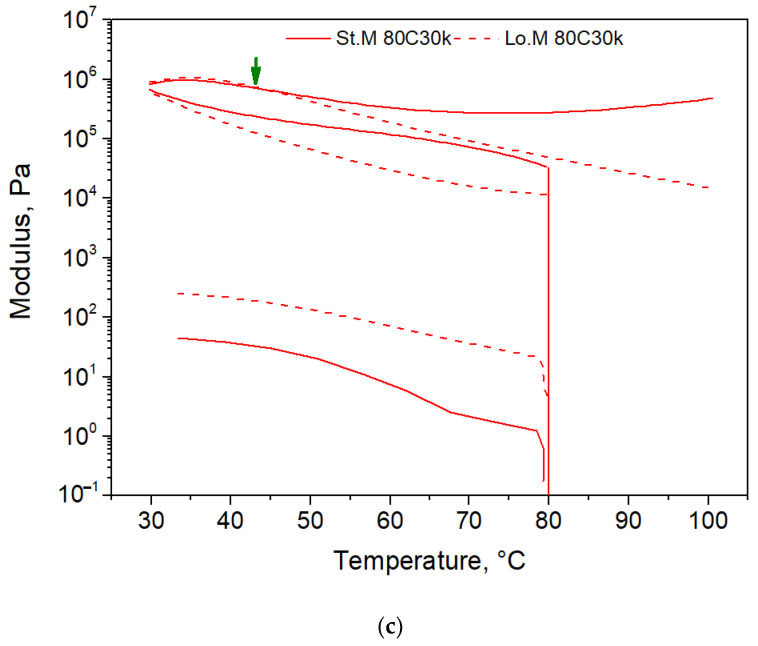
(**a**). Change in storage and loss moduli of preE after being cured to achieve 30 kPa at 60 °C, then cooling to 30 °C and heating to 100 °C. (**b**). Change in storage and loss moduli of preE after being cured to achieve 30 kPa at 70 °C, then cooling to 30 °C and heating to 100 °C. (**c**) Change in storage and loss moduli of preE after being cured to achieve 30 kPa at 80 °C, then cooling to 30 °C and heating to 100 °C, (*) the loss modulus overtakes the storage modulus during cooling from testing temperature to room temperature, (arrow) the storage modulus overtakes the loss modulus during heating from 30 °C to 100 °C.

It is clearly seen that the values of 20 kPa, 30 kPa and 40 kPa were the storage moduli at the pre-cured temperatures of 60 °C, 70 °C and 80 °C, respectively. Therefore, the moduli at room temperature would be higher. Furthermore, it is essential to investigate the behavior of preE versus temperature for the foaming process. Figure 2 shows the pre-curing of epoxy resin to achieve preE with a storage modulus of 30 kPa (at 60 °C, 70 °C and 80 °C); then it was cooled down to 30 °C and heated again to 100 °C. At the resin stage, the storage modulus (solid line) was lower than the loss modulus (dashed line). After pre-curing to 30 kPa, the storage modulus was higher than the loss modulus for all samples pre-cured at 60 °C (Figure 2a), 70 °C (Figure 2b) and 80 °C (Figure 2c). During cooling down to 30 °C, the storage modulus and loss modulus crossed over for 60C30k and 70C30k (*) but not for 80C30k. This implied that both preE 60C30k and preE 70C30k showed more viscoelastic behavior as compared to preE 80C30k at room temperature (30 °C). 

When being heated from 30 °C to 100 °C, preEs exhibited a distinctive pattern between storage and loss moduli. The storage modulus was reduced with higher temperatures but then increased further, while the loss modulus continuously decreased with higher temperatures. This behavior is completely different from a thermoplastic and a fully cured epoxy. The first distinguishing characteristic of preE was the crossover point between the storage modulus and loss modulus at a specific temperature—T_cross_ (the arrow in Figure 2a–c). It reflects that preE changed its behavior from viscous-dominated to elastic-dominated behavior because of further crosslinking reactions. The second distinguishing characteristic of preEs was that the storage modulus started to increase at a certain temperature—T_low_ (the red star). It implied that further crosslinking reactions occurred and resulted in a shorter *M_c_* of the preEs. 

*M_c_* is a common term to represent the crosslinked network structure. Put simply, 1st stage—epoxy and hardener react to form dimers, trimers, oligomers; 2nd stage—oligomers react with each other to form one network and a gel structure is formed, but reactive end groups are still present; 3rd stage—when the preE achieves a full degree of cure, only one three-dimensional (3D) crosslinked network is achieved. This research fabricated a preE of the 1st stage (60C20k), and various preEs after the 2nd stage. Hence, preE 60C20k showed viscous-dominated behavior as compared to other preEs because it did not reach the gel point. The other preEs presented elastic-dominated behavior, or plastic-dominated behavior depending on their pre-curing history and the applied foaming temperature. This could be observed from the storage modulus of preE at 30 °C (St.M_30 °C_), as seen in Table 2. The low value of 60C20k was a value before the gel point, and the low value of 70C20k was a value just 0.3 min after gel time. All the preEs cured at 80 °C had high values of the storage modulus at 30 °C. 

The rheological properties of preEs from Figure 2 are displayed in Table 2. Firstly, T_cross_ decreased from 60C20k to 60C30k to 60C40k, while all the preEs pre-cured at 70 °C and 80 °C had similar T_cross_. The results reflected the pre-curing time well (as seen in Table 1), where 60C20k was pre-cured before the gel point as compared to 60C30k and 60C40k. However, the storage moduli at the cross point (St.M_crs_) were definitely different for every preE; meaning that they had completely different pre-crosslink networks. Secondly, the temperature (T_low_) at the lowest storage modulus of preEs increased slightly in the 60 °C series and 70 °C series but was similar in the 80 °C series. Furthermore, there was a similar T_low_ for 60C20k versus 70C20k, 60C30k versus 70C30k and 60C40k versus 70C40k versus all preEs at 80 °C. This result implied that preEs with a higher degree of cure of pre-crosslink network need a higher temperature to activate further crosslink reactions. It was assumed that foaming preE at T_low_ values of 70–80 °C could provide the lowest density because preE had the most mobility of the pre-crosslink network at these temperatures. It is noted that the St.M_low_ of all preEs was discerned. The optimum range of the storage modulus for the foaming window was estimated and is reported in the next section. 

### 3.2. Glass Transition Temperature and Onset Temperature of Further Curing of PreE 

**Figure 3:**
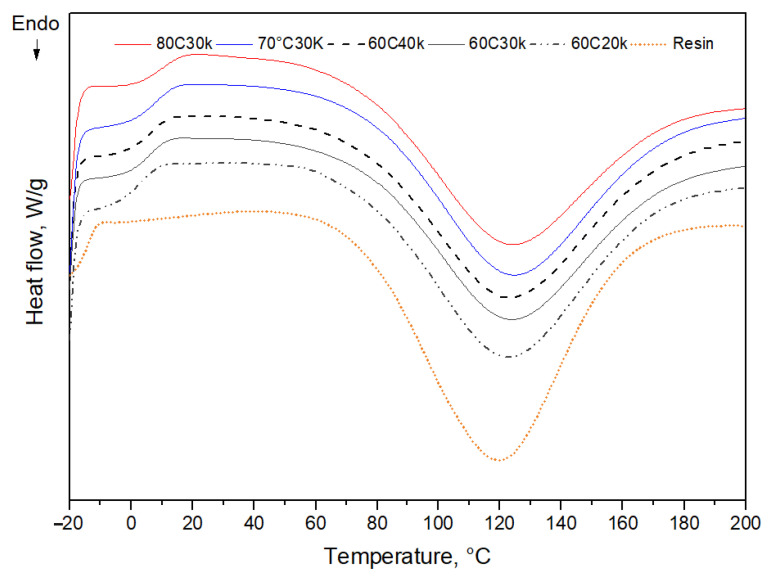
Thermal properties from DSC of preEs showing glass transition and further curing.

Thermal properties of preEs are shown in Table 3 and Figure 3. All the preEs had a T_g_ below 25 °C (room temperature). The T_g_ of preEs are as expected and reflect the pre-curing history well. A preE with a higher storage modulus and higher curing temperature has a higher T_g_. That trend in T_onset_ of the further curing process of preE agrees with the trend in T_cross_ rheology results, as shown in Figure 2. In detail, T_onset_ values obtained by DSC are slightly higher than T_cross_ obtained by the rheology test. In fact, the sample used in DSC was only about 10 mg and experienced only the thermal process, while the sample in rheology was almost 2 g and experienced both thermal and oscillating shear. The rheology sample was thus cured slightly faster. It is observed that preE 60C30k had a slightly higher curing percentage as compared to 70C20k. This result is also confirmed with *M_c_* (measured by a swelling method). This is because 60C30k was cured in 47.5 min while 70C20k was cured in 27.5 min. 

### 3.3. Molecular Weight between Crosslinks by Solvent Method 

The gel fraction is based on the absorption of acetone (CH_3_-CO-CH_3_) into the pre-crosslinked network of preEs. The reaction between the epoxy group of DGEBA and H-active of amine groups depends greatly on the curing temperature. Based on steric hindrance, the H-active from the primary amine reacts with the epoxy ring twice as fast as that of the secondary amine [8,25]. By changing the curing temperature, the reaction rate can be adjusted and the pre-crosslinked network of preE can be varied. In addition, changing the curing time controls the degree of cure and the gel state of preE. As seen in Table 4, 60C20k (before gel point) had the highest *M_c_*, whilst 80C40k had the lowest *M_c_*. Therefore, pre-curing temperature had a key effect on the value of *M_c_* of preE from DGEBA and 260A. 

**Figure 4:**
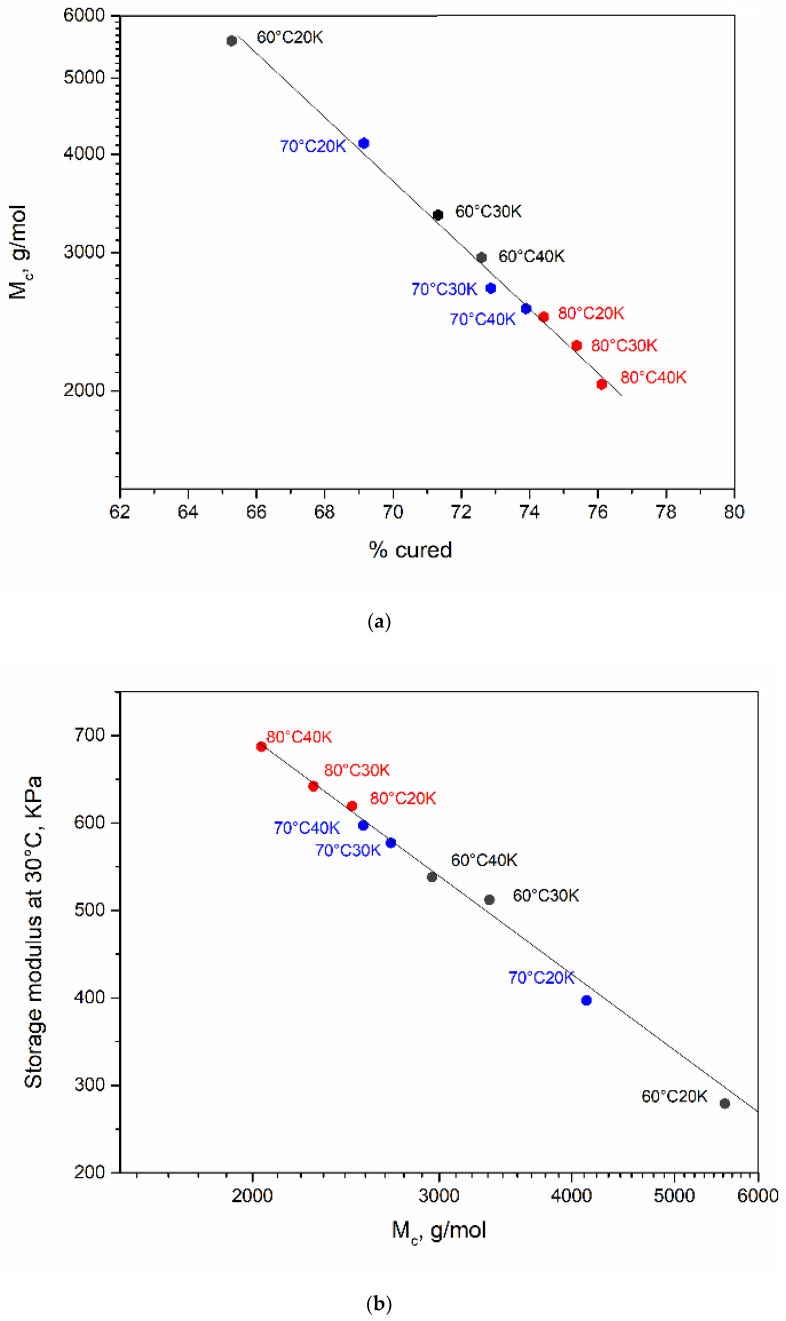
(**a**) Relationship between the degree of cure and *M_c_*. (**b**) Relationship between *M_c_* and the storage modulus at 30 °C, St.M_30 °C_ of preE.

At the same pre-curing temperature, the longer the curing time, the higher the modulus of preE, leading to lower *M_c_*. It is important to note that preEs with the same value of storage modulus but different temperatures showed different *M_c_* (such as 60C20k, 70C20k and 80C20k). Therefore, *M_c_* versus degree of cure, and *M_c_* versus storage modulus of preEs at room temperature, were plotted and are shown in Figure 4. 

The logarithmic plot of degree of cure and *M_c_* are displayed in Figure 4a, and that of *M_c_* and storage modulus at room temperature can be seen in Figure 4b. It was found that preE 60C30k (curing time at 60 °C—47.5 min) had a higher degree of cure and lower *M_c_* and a higher St.M_30 °C_ as compared to 70C20k (curing time at 70 °C—27.5 min). Therefore, the effect of longer curing time on *M_c_* was obvious. Figure 4 proves that the degree of cure, the *M_c_* and the St.M_30 °C_ are designable with a specific curing temperature and a specific curing time.

### 3.4. CO_2_ Absorption Content of PreE 

All preEs have almost similar CO_2_ concentrations after 5 h of impregnation in the autoclave at a pressure of 55 bar (as shown in Table 4). Similar findings were also reported by Ito et al. (2014). CO_2_ concentration depends on the crosslinked network and chemical structure of epoxy and hardener. As all preEs were prepared from DGEBA and 260A, CO_2_ could be absorbed similarly into the free volume of all the pre-crosslinked networks. 

### 3.5. Effect of Foaming Temperature on Density of Epoxy Foams Prepared by Solid-State Foaming Technique

**Figure 5:**
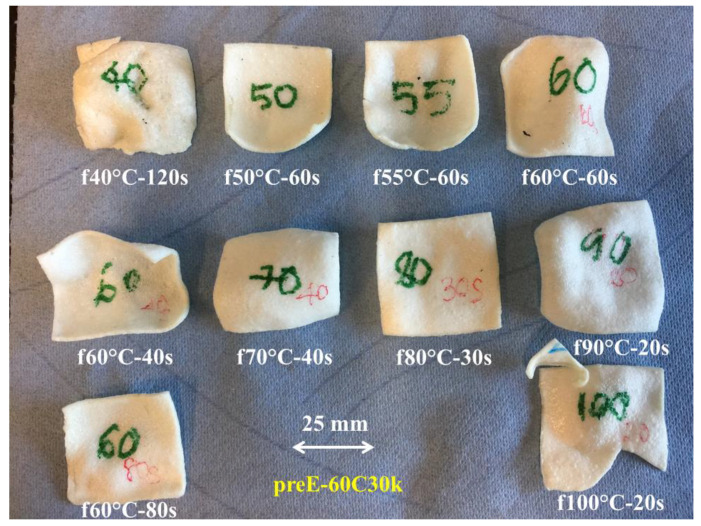
Foaming of preE 60C30k at different foaming temperatures and times.

Figure 5 shows the epoxy foam from the solid-state CO_2_-foaming technique of the preE 60C30k, which had the lowest density among all the preEs. The preE was able to expand at all temperatures from 40 °C to 100 °C with different foaming times. This was free expansion in an oven, which is only workable for preEs in a solid state. After heating, the pre-crosslinked network of the preE regains mobility which ensures expansion or CO_2_ (as a very small molecule compared to the pre-crosslink network) is easily diffused out of the pre-crosslink networks. As discussed above, the preE behaves differently as compared to thermoplastics because of its further curing ability. Therefore, a low foaming temperature led to low mobility of the pre-crosslink network of preE and also low pressure of CO_2_ and a low diffusion rate. At higher temperatures (70–80 °C), the pre-crosslink network had the lowest storage modulus and also started to crosslink further, and the pressure of CO_2_ was also higher. At foaming temperatures of 90 °C and 100 °C, the pre-crosslinked network started to cure further and the pressure of CO_2_ was very high. Each preE had different pre-crosslink networks and different moduli, hence the foaming ability and the cell morphology were different. 

All the preEs were different not only in *M_c_* and T_g_ but also in modulus at room temperature. Furthermore, the modulus of preE not only decreases with temperature but also increases because of further curing. This study focused on the change in storage modulus and loss modulus with foaming temperature and their effect on the foaming of preE (called the solid-state foaming technique). Therefore, foaming temperature was varied in 10 °C steps from 40 °C to 100 °C. There were nine preEs and seven foaming temperatures, so there were sixty-three samples in total. The lowest density at each foaming temperature is shown in Table 5. This is because preEs showed viscoelastic behavior during foaming. They could expand and then shrink depending on their pre-crosslinked network (as shown in Figure 5, a foaming temperature of 60 °C for 80 s caused shrinking as compared to 40 s and 60 s). This behavior’s effect on the cell morphology is discussed in the next section.

**Figure 6:**
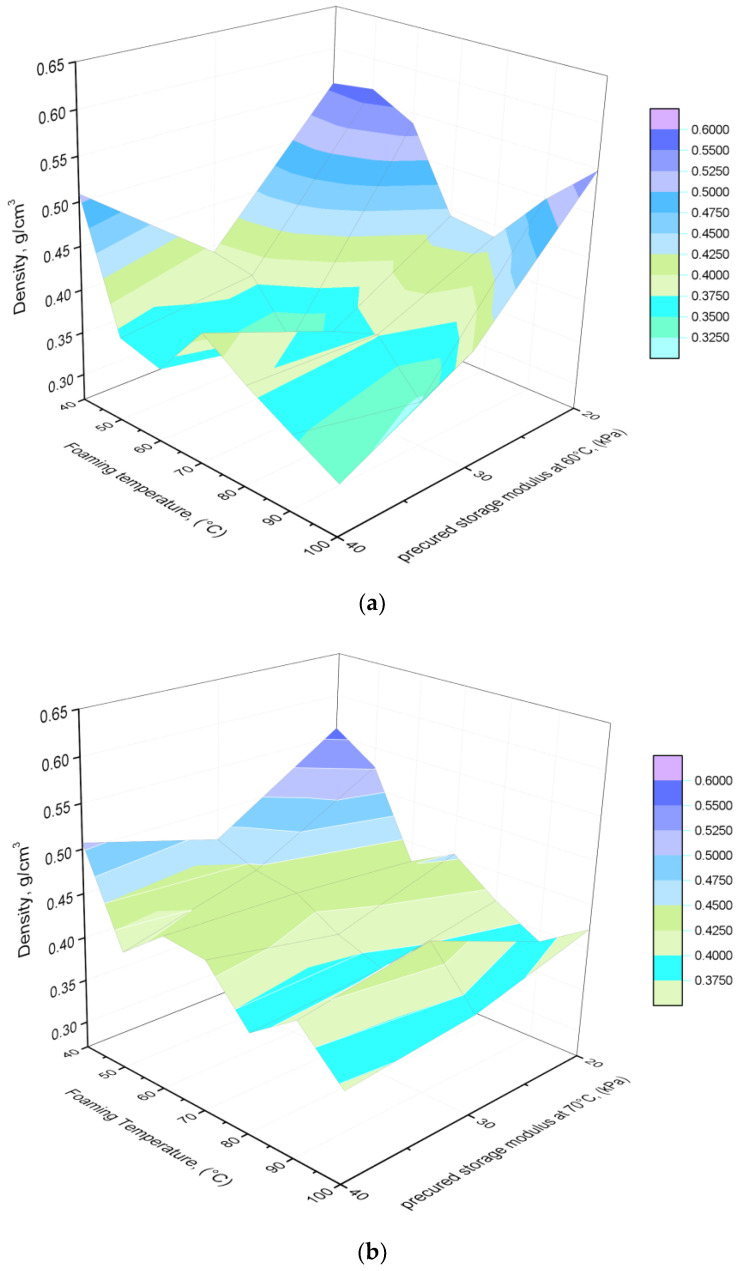

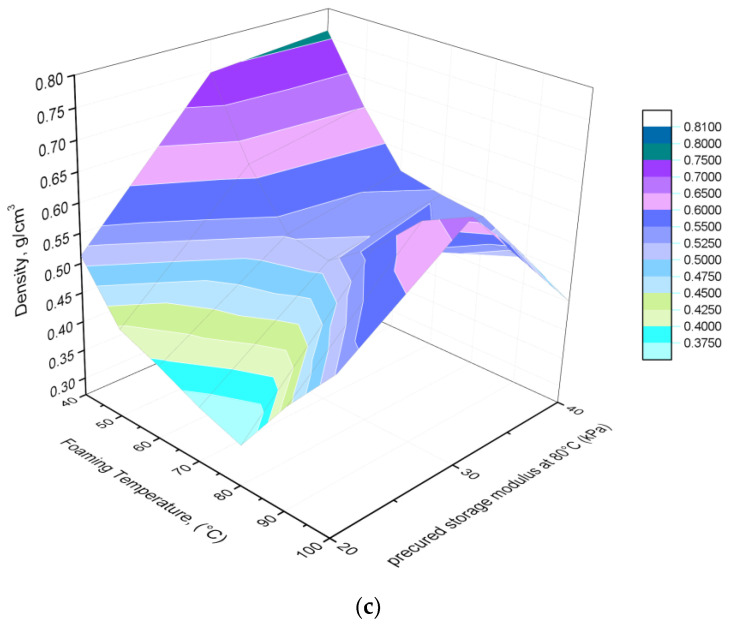
(**a**) Density range of epoxy foam from preE 60 °C series foaming at 40 °C to 100 °C. (**b**) Density range of epoxy foam from preE 70 °C series foaming at 40 °C to 100 °C. (**c**) Density range of epoxy foam from preE 80 °C series foaming at 40 °C to 100 °C (the axis of pre-cured modulus has the order of 20 k, 30 k, 40 k of (**c**), which is different from Figure 6a,b for a better view).

Figure 6 shows the density of different preE series. The *x*-axis shows the foaming temperature (40–100 °C), the *y*-axis shows the pre-cured storage modulus (20–40 kPa) and the *z*-axis presents the density of solid-state epoxy foam. In this solid-state foaming technique, both factors of the pre-crosslinked network and the pressure of CO_2_ will control the expansion of preE. The discussion is focused on density values below 0.45 g/cm^3^. 

PreE pre-cured at 60 °C (60C20k) shows a pre-cured network with low crosslink density. Large oligomeric structures were still present. As a result, CO_2_ was readily diffused out of the network, resulting in low expansion rates and high density of the obtained epoxy foam, as supported by Figure 6a. The 60C20k sample could achieve densities below 0.45 g/cm^3^ if it was foamed at temperatures of f70 °C and f80 °C. These temperatures are close to the value of T_low_ in Table 2, at which the storage modulus starts to increase and stabilizes the foam structure and prevents the diffusion loss of CO_2_. In the other case, preE 60C30k achieved a density below 0.40 g/cm^3^ at all the foaming temperatures (f40–f90 °C). Therefore, it is assumed that 60C30k with *M_c_* of 3348.1 g/mol was the most expandable pre-crosslinked network. Similarly, preE 60C40k exhibited a density below 0.40 g/cm^3^ when being foamed from f50–f100 °C except at f70 °C. PreE 60C40k had a high value of St.M_low_ (106.7 kPa) at T_low_ of 74.37 °C, as shown in Table 2. Hence, it could exhibit elastic-dominant behavior, causing the shrinkage. Beyond 70 °C, f80–f100 °C would provide higher CO_2_ pressures to expand preE 60C40k. 

The preE 70 °C series (20 k, 30 k, 40 k) revealed a density range of 0.37–0.56 g/cm^3^ as compared to a range of 0.32–0.56 g/cm^3^ of the preE 60 °C series (as displayed in Table 5). It is noted that all the pre-crosslinked networks in preE 70 °C series were cured after the gelation stage. The lowest density was obtained at the high foaming temperatures (f80 °C, f90 °C and f100 °C). This originated from further curing at these temperatures which stabilized the cellular structure during the foaming process. As expected, the different foaming behaviors between the preE 70 °C series and preE 60 °C series would be inherited by the cell morphology. 

Predictably, the preE 80 °C series expanded to a higher density range of 0.34–0.76 g/cm^3^. Only the pre-crosslinked network of 80C20k (*M_c_* of 2483.9 g/mol) could still expand to achieve density below 0.40 g/cm^3^ (at f60–f80 °C). Both preE 80C30k and 80C40k had density of above 0.50 g/cm^3^ because they had more crosslinks via their *M_c_* values of 2283.9 g/mol and 2039.0 g/mol, respectively. The dense pre-crosslinked network provided a high storage modulus, less mobility and resistance to expansion. In a brief conclusion, the *M_c_* in the range of 2400–4200 g/mol showed expansion to densities below 0.45 g/cm^3^. 

### 3.6. Morphology of Epoxy Foams 

**Figure 7:**
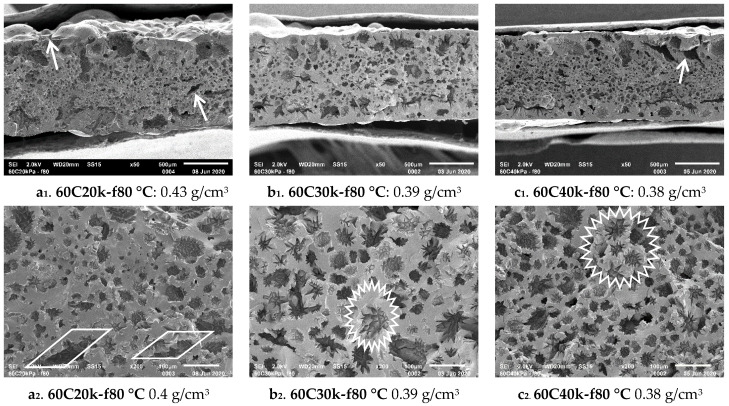
Morphology of epoxy foam fabricated from the solid-state CO_2_ foaming at f80 °C of preE 60 °C series.

Epoxy foams prepared from the solid-state foaming technique exhibited various cell morphologies, because of the difference in *M_c_* and storage modulus of preEs. According to Figure 2a–c and Table 2, the storage moduli of all of preEs were the lowest at temperatures of 71.3–74.4 °C. This is where preE had the most mobility of the pre-crosslink network. Therefore, the foam samples at foaming temperatures of f60 °C and f80 °C were selected to check the SEM morphologies. Figure 7a_1_–c_1_ display the SEM at 50X magnification, while Figure 7a_2_–c_2_ display the SEM at 200X magnification of the preE 60 °C series. Predictably, 60C20k was torn easily at the surface by CO_2_ pressure because 60C20k had not reached the gel stage (Figure 7a_1_,a_2_). Epoxy foam of 60C30k-f80 °C shows large star polygons and some irregular cells (Figure 7b_1_,b_2_). Epoxy foam from 60C40k-f80 °C has many star polygon cells and also some cracks (Figure 7c_1_,c_2_), which are smaller than those of 60C30k. The star polygon is the rebound form of a spherical shape due to the elastic characteristics of the preE 60 °C series (having *M_c_* in the range of 2956–5577 g/mol). 

**Figure 8:**
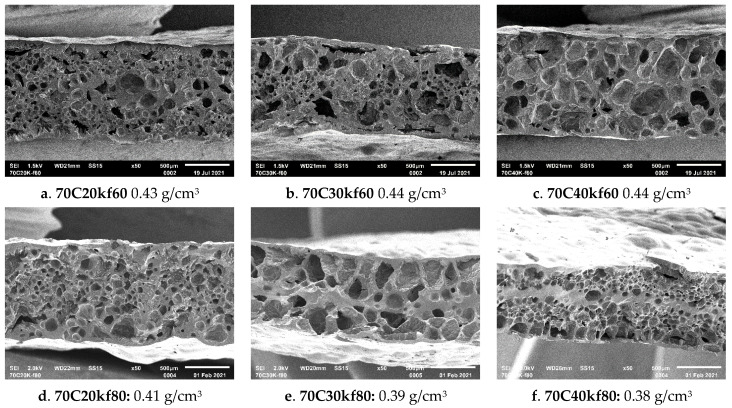
Morphology of 50X magnification of epoxy foam fabricated from the solid-state CO_2_ foaming at f60 °C and f80 °C of preE 70 °C series.

**Figure 9:**
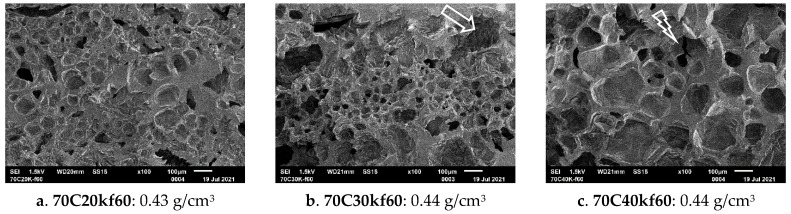

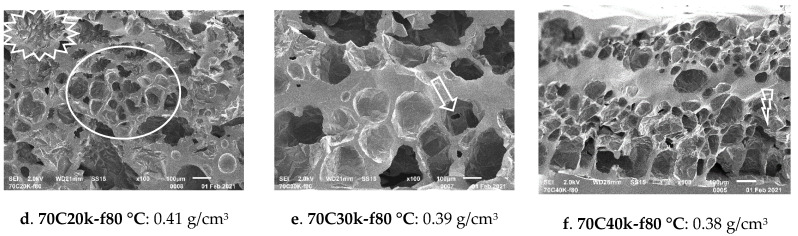
Morphology of 100X magnification of epoxy foam fabricated from the solid-state CO_2_ foaming at f60 °C and f80 °C of preE 70 °C series.

The cell morphology of epoxy foam in the preE 70 °C series is obviously different. More spherical cells were observed in the preE 70 °C series. Figure 8a–f display the SEM at 50X magnification. The effect of f60 °C and f80 °C on the foam morphology was less different in 70C20k (Figure 8a versus Figure 8d), and more different in 70C30k (Figure 8b versus Figure 8e) and 70C40k (Figure 8c versus Figure 8f). According to Figure 4a,b, *M_c_* of 70C20k was the second highest (even higher than that of 60C30k). Therefore, the *M_c_* of 70C20k (4130.8 g/mol) was more expandable and had more small spherical cells in 70C20k-f60 °C (Figure 9a) and 70C20k-f80 °C (Figure 9d) as compared to 70C30k and 70C40k. The 70C20k-f80 °C sample (Figure 9d) had fewer star polygon cells and more spherical cells. 

PreE 70C30k and 70C40k reflect the effect of foaming temperature and the storage modulus of preEs well. In comparison, 70C30k-f60 °C (Figure 8b) had a lower CO_2_ pressure at 60 °C, hence the expanded cells were small. The 70C30k-f80 °C sample (Figure 8e) had a higher CO_2_ pressure, and the *M_c_* was short enough to hold the expanding CO_2_, so large cells were produced. This was similar to 70C40k-f60 °C, which also had a small *M_c_* to produce large expanded cells. However, the cells became smaller and there was also a solid line in the center of 70C40k-f80 °C (Figure 8f). This means that 70C40k-f80 °C started to cure further and increased its *M_c_* at 80 °C to expand less. 

At a high magnification of 100X, 70C30k-f60 °C (Figure 9b) shows micron cells and some large cells with irregular shapes. However, 70C30k-f80 °C has more and larger spherical cells and also some interconnected cells (Figure 9e). A cracking of the thin wall is shown in 70C40k-f60 °C (Figure 9c). Cell wall stretching and rupture were observed in 70C40k-f80 °C (Figure 9f). It is concluded that the pre-crosslinked structure of 70C30k and 70C40k showed less elastic- and plastic-dominated behavior to maintain the spherical cells without shrinkage to form star polygons cells. Theoretically, pre-curing at a higher temperature provides higher energy to activate the secondary amine to react with epoxy. Hence, the preE 70 °C series has more branching and more crosslink networks as compared to the preE 60 °C series. 

**Figure 10:**
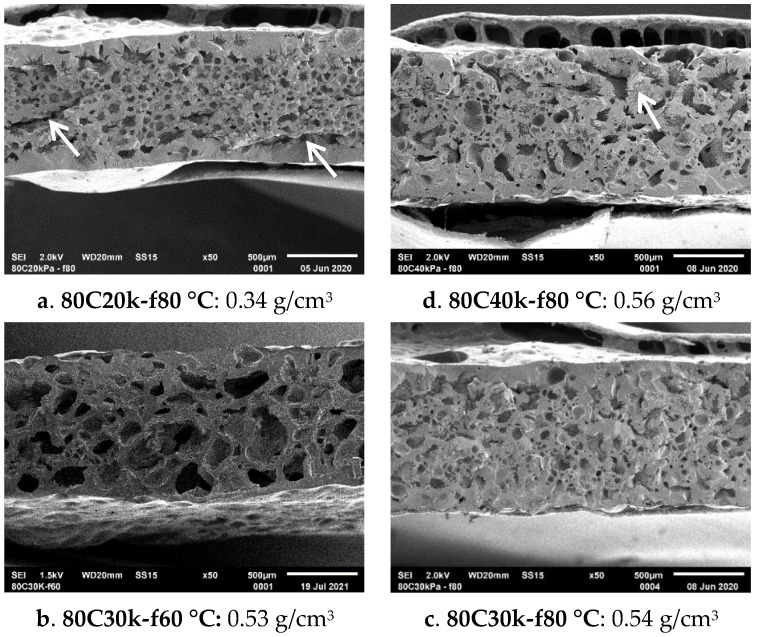
Morphology of 50X magnification of epoxy foam fabricated from the solid-state CO_2_ foaming at f60 °C and f80 °C of preE 80 °C series.

According to the density result of the preE 80 °C series, as shown in Figure 6c, 80C20k has densities below 0.45 g/cm^3^ when foamed at f50–f90 °C. As observed, 80C20k-f80 °C (Figure 10a) possesses smaller cells and some long cracks as compared to the others. Both 80C30k-f80 °C (Figure 10c) and 80C40k-f80 °C (Figure 10d) show more irregular opened crack shapes. Their low *M_c_* values restrain the expansion. Furthermore, 80C30k and 80C40k would start to cure further at f80 °C and obstruct the expansion. In addition, the effect of foaming temperature on morphology is present in 80C30k-f60 °C (Figure 10b) and 80C30k-f80 °C (Figure 10c). A lower foaming temperature induced a lower CO_2_ pressure to foam. Hence, 80C30k-f60 °C was produced with large cells and thick walls, as shown in Figure 10b. 

**Figure 11:**
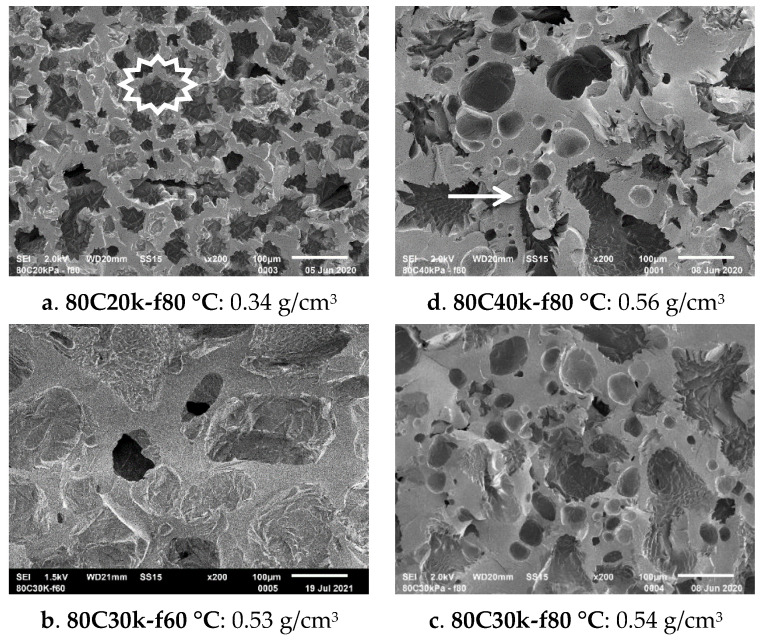
Morphology of 200X magnification of epoxy foam fabricated from the solid-state CO_2_ foaming at f60 °C and f80 °C of preE 80 °C series.

Observed from Figure 11a, the cell morphology of 80C20k-f80 °C also shows star polygons. However, its star polygon cells were larger and had thicker walls. This means 80C20k presented slightly less elastic behavior and less shrinkage. The cell morphologies of 80C30k-f80 °C and 80C40k-f80 °C are irregular and spherical, as seen in Figure 11c,d, respectively. There was also cracking of the thick walls. Their cell morphologies originated from the high storage modulus and the low *M_c_* (in the range of 2039–2284 g/mol), and showed more plastic deformation during foaming. Focusing on the effect of foaming temperature, 80C40k-f60 °C (foamed at 60 °C) had a very thick wall of large cells and no microcells, as shown in Figure 11b; while 80C40k-f80 °C (being foamed at 80 °C) had more small cells, as seen in Figure 11c. 

This study found an important point, that epoxy foam from 80C20k-f80 °C (Figure 11a) had a star polygon cell structure even though its *M_c_* was lower than that of 70C30k-f80 °C (Figure 9e) and 70C40k-f80 °C (Figure 9f). Hence, using only *M_c_* could not represent the foaming behavior of preE because this value will be changed due to further curing and it is impossible to measure. The storage modulus at foaming temperatures (which can be estimated by rheology) is more suitable to be used in the solid-state foaming technique of epoxy pre-crosslinked networks.

### 3.7. The Role of Storage Modulus in the Solid-State CO_2_-Foaming Technique 

**Figure 12:**
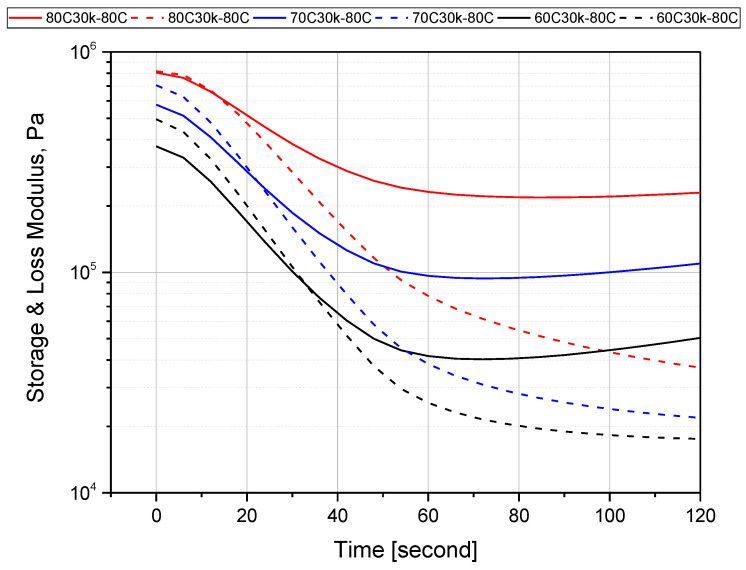
Storage modulus (solid) and loss modulus (dash) of preE 60C30k, 70C30k and 80C30k at 80 °C.

**Figure 13:**
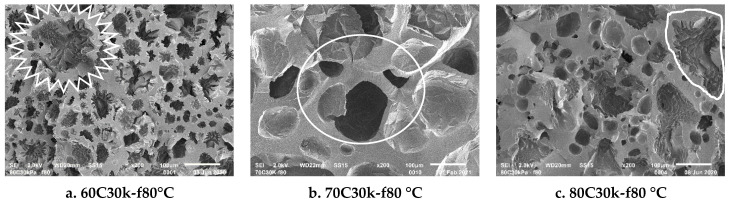
Distinguishing cell morphology of 60C30k, 70C30k and 80C30k with different storage moduli at a foaming temperature of 80 °C.

The cell morphology of preE 60C30k, 70C30k and 80C30k was examined by using their viscoelastic, elastic-dominated and plastic-dominated characteristics via the storage modulus and loss modulus at 80 °C. Firstly, the DGEBA–260A was cured to a storage modulus of 30 kPa; secondly, the preE was cooled down to 30 °C; lastly, preE was immediately heated and held at 80 °C. Figure 12 shows the storage modulus at 80 °C of preE with different holding times. This information was used to estimate the storage modulus of preE at a foaming temperature of 80 °C. From 0–30 s, the storage modulus of 60C30k was lower than its loss modulus. PreE 60C30k showed strong damping and viscous-dominated behavior. Therefore, its spherical cells shrank and became star polygons, as seen in Figure 13a. In the case of 70C30k, the storage and loss moduli were higher than those of 60C30k and both moduli crossed each other after 20 s. This means the pre-crosslinked structure of 70C30k showed low damping and plastic-dominated behavior. During the foaming process, the expanding cells were stable in a spherical shape and interconnected, as shown in Figure 13b. PreE 80C30k shows that its storage and loss moduli were the highest among the three preEs. Both moduli crossed each other after a few seconds, showing high plastic behavior. Hence, 80C30k expansion was difficult and, therefore, produced irregular cell morphologies. The irregular cells were shallow and also had no interconnections, as seen in Figure 13c. 

### 3.8. Proposal of the Relationship between Storage Modulus at Foaming Temperature and Density 

Figure 12 shows that the most critical factor affecting the density of epoxy foam (prepared by solid-state foaming) is the storage modulus. Moreover, the temperature dependence of the storage modulus of preE is complex. As seen from Figure 2, the storage modulus changes parabolically with temperature due to further curing. This means the storage modulus also changes during free expansion based on the specific foaming temperature. During free expansion in an oven, the heat is transferred to the top surface of the preE sheet by convection of hot air and to the bottom of the preE sheet by conduction of the metal plate. 

**Figure 14:**
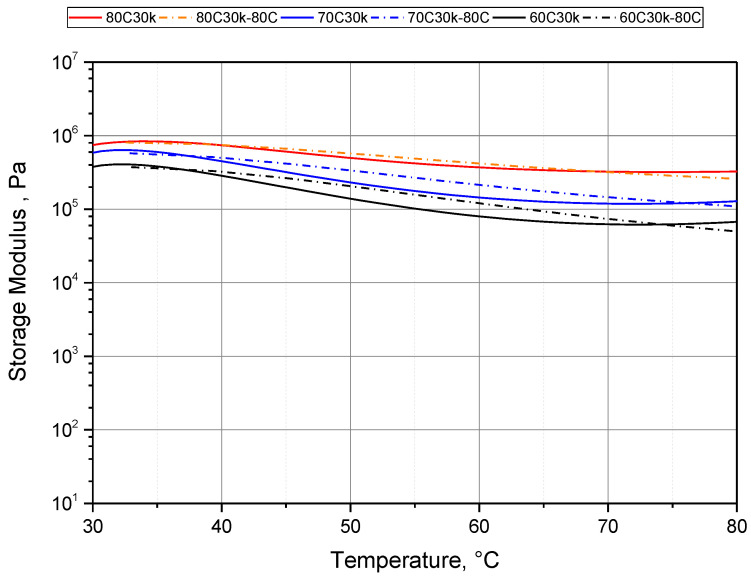
Storage modulus of preEs of 60C30k, 70C30k and 80C30k at 80 °C (dash-dotted line) and their storage modulus at a heating rate of 10 °C/min (solid line).

There are two options to measure the storage modulus from the rheology test, isothermal at a specific temperature and dynamic thermal in a temperature range with a specific heating rate. PreEs of 60C30k, 70C30k and 80C30k were selected to test the two routes. The storage moduli from the two testing routes are shown in Figure 14. The solid curves are the storage moduli of preEs heated from 30 °C to 100 °C with a 10 °C/min heating rate. The dash-dotted curves highlight the storage modulus of preEs heated at an isothermal of 80 °C. Both storage moduli showed a decreasing trend with time. The differences in 70C30k and 60C30k were more pronounced at 50 °C and 60 °C. The difference in both moduli for 80C30k was small. Hence, it is reasonable to use of the value of the storage modulus (measured from a dynamic test scanning from 40 °C to 100 °C) to estimate the storage modulus of the preEs during foaming at a temperature of f40 °C to f100 °C, respectively. 

**Figure 15:**
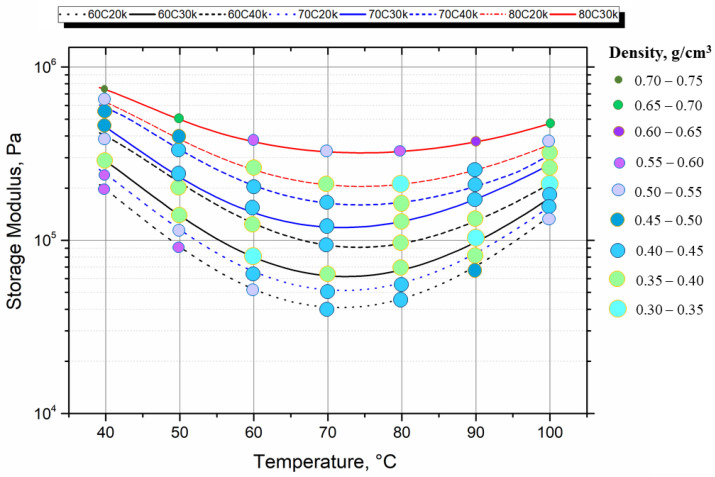
Storage modulus vs. temperature as the impacts on density of epoxy foam from preE.

The relationship between the storage modulus and temperature of preE is summarized in Figure 15. The resulting foam density is indicated by different sizes and colors of the symbols. As can be seen, an optimum foaming window is found for storage moduli between 6 × 10^4^ and 2 × 10^5^ Pa, and for temperatures between 60 °C and 80 °C. Here, the DGEBA–260A epoxy system can be foamed to densities lower than 0.45 g/cm^3^ (light blue and light green circles).

This minimum foam density is affected by the complex interplay between the temperature, the storage modulus and the diffusion and the pressure of CO_2_ (assumed to obey the gas ideal equation PV = nRT). It is noted that the optimum storage modulus at the foaming temperature would prevent the diffusion of absorbed CO_2_ at that temperature. At an expansion temperature below 60 °C, the storage modulus of preE was high and the CO_2_ pressure was low, so a higher density was produced. The optimum expanding temperature range was 60–80 °C. At these optimum temperatures, the storage modulus of preEs was the lowest and their absorbed CO_2_ pressure was higher. Therefore, a density was achieved below 0.45 g/cm^3^. Free expansion at 90 °C and 100 °C was able to achieve a density below 0.45 g/cm^3^ only for certain preEs. In detail, 70C20k, 60C30k and 60C40k (*M_c_* in the range of 2900–5600 g/mol) were expanded below 0.45 g/cm^3^ at 90 °C. Meanwhile, 60C40k, 70C30k and 70C40k (*M_c_* in the range of 2500–2900 g/mol) were expanded below 0.45 g/cm^3^ at 100 °C. This means free expansion to a low density needs an optimum window of storage modulus and CO_2_ pressure. In summary, pre-curing of the epoxy system can control the elasticity (via storage modulus) and thus the foam expansion behavior. 

## 4. Conclusions

Controlled foaming of epoxy by using physical CO_2_ gas was developed by using the solid-state CO_2_-foaming technique. This study demonstrated that preE from DGEBA–260A with the storage modulus in the range of 20–40 kPa at a specific curing temperature achieved solid-state behavior at room temperature. These solid-state pre-curing epoxy preEs are expandable with CO_2_ gas. All preEs exhibited further curing and had their lowest storage modulus (60–200 kPa) at temperatures of 70 °C and 80 °C. These temperatures were also the optimum foaming temperatures for most of the preEs to achieve a density of 0.32–0.45 g/cm^3^. The solid-state CO_2_-foaming technique can be applied for various epoxy and hardener systems. However, the difference in chemical structures of epoxy and hardener could result in a different foam processing window.

## Figures and Tables

**Table 1:** Curing time (minutes) to achieve different storage moduli and gel points based on rheology at 60 °C, 70 °C and 80 °C.

		20 kPa		30 kPa	40 kPa
60 °C	-	45.0	*46.0-gel*	47.5	48.7
70 °C	*27.2-gel*	27.5	-	29.7	30.3
80 °C	*14.0-gel*	15.0	-	15.3	15.6

**Table 2:** Rheology properties of preEs that underwent heating from 30 °C to 100 °C at 10 °C/min.

	60C20k	60C30k	60C40k	70C20k	70C30k	70C40k	80C20k	80C30k	80C40k *
St.M_30 °C_, kPa	279.0	512.0	538.0	397.0	577.0	597.0	619.0	642.0	687.0
T_cross_, °C	53.9	52.8	48.7	47.3	47.3	47.3	45.5	44.5	-
St.M_crs_, kPa	71.4	115.8	249.9	264.4	268.6	383.9	475.0	621.5	-
T_low_, °C	71.3	72.3	74.4	71.8	72.4	74.4	74.4	74.4	-
St. M_low_, kPa	40.9	61.8	106.7	118.5	138.5	160.0	199.6	319.6	-

* 80C40k: data could be not obtained due to machine limitation, St.M_30 °C_: the storage modulus of preE after cooling down to 30 °C, T_cross_: the temperature at cross point between storage and loss moduli, St.M_crs_: the storage modulus of preE at the cross point with loss modulus and St.M_low_: the lowest storage modulus during further heating from 30 °C to 100 °C.

**Table 3:** T_g_ and further curing properties of preEs.

	Resin	60C20k	60C30k	60C40k	70C20k	70C30k	70C40k	80C20k	80C30k	80C40k
T_g_, °C	−11.3	3.6	6.3	6.4	7.7	7.7	8.2	9.3	9.5	13.9
T_onset,_ °C	58.1	56.2	52.1	51.1	57.2	55.7	55.5	48.3	51.9	52.2
% cure	-	65.27	71.32	72.59	69.15	72.87	73.89	74.42	75.38	76.11

% cure: curing percentage of preE.

**Table 4:** Molecular weight between crosslinks (*M_c_*, g/mol), and the CO_2_ content after 5 h absorption of preE.

	60C20k	60C30k	60C40k	70C20k	70C30k	70C40k	80C20k	80C30k	80C40k
*M_c_*	5577.5	3348.1	2956.1	4130.8	2701.5	2544.6	2483.9	2283.9	2039.0
CO_2_ %	10.75	11.19	11.28	11.21	12.17	11.41	11.27	11.15	11.08

**Table 5:** Density (g/cm^3^) of epoxy foams from various preEs at different foaming temperatures.

	f40 °C	f50 °C	f60 °C	f70 °C	f80 °C	f90 °C	f100 °C
60C20k	0.56	0.56	0.54	0.44	0.43	0.50	0.55
60C30k	0.39	0.38	0.33	0.36	0.39	0.32	0.40
60C40k	0.51	0.37	0.35	0.42	0.38	0.35	0.33
70C20k	0.56	0.57	0.43	0.45	0.41	0.39	0.42
70C30k	0.46	0.45	0.44	0.41	0.39	0.44	0.38
70C40k	0.51	0.40	0.44	0.44	0.38	0.42	0.37
80C20k	0.51	0.42	0.39	0.36	0.34	0.44	0.53
80C30k	0.75	0.62	0.53	0.51	0.56	0.64	0.67
80C40k	0.76	0.65	0.56	0.55	0.54	0.50	0.45
